# Parents’ digital skills and their development in the context of the Corona pandemic

**DOI:** 10.1057/s41599-023-01556-7

**Published:** 2023-03-11

**Authors:** Badr A. Alharbi, Usama M. Ibrahem, Mahmoud A. Moussa, Mona A. Alrashidy, Sameh F. Saleh

**Affiliations:** 1 University of Ha’il, Hail, Kingdom of Saudi Arabia; 2 Faculty of Education, Suez Canal University, Ismailia, Egypt

**Keywords:** Education, Science, technology and society

## Abstract

We investigate parents’ and guardians’ digital skills and the extent of their development in the context of the spread of the Corona epidemic. In addition, we sought to explore the differences in digital skills between parents and their employment status, age, and responsibility in teaching children. We sought to rely on the descriptive-analytical approach and prepared a scale of eight theoretical dimensions with the participation of 250 students’ Saudi parents. The application of the study was by online submission form (via Edit Submission). Our findings showed that there was a discrepancy in the performance of the sample, which was very high in the dimensions of operational skills, instrumental skills, and cognitive constructivism skills. There were also differences between the effect of computers on the instrumental skills and cognitive constructivism skills of the parents. Parents’ dependence on alternative digital sources in exploring for information, formulating knowledge, manipulating it, and criticizing. The learner can reach the cognitive level in a more flexible manner, which allows him to gain learning objectives. The knowledge navigation can be developed because of different online outdoor exercises and software familiar. This requires self-organization to search for appropriate knowledge to use in the renewal of the cognitive structure.

## Introduction

Students, teachers, parents, and other people with a stake in higher education are paying attention to developing digital skills because it is one of the most important skills for work and school. Conclusions about digital skills showed how digital technologies could support easy access to data and enhance social status (Passey and Tatnall, 2014). In addition, digital technology has changed the form of communication with others, ways of accessing information, navigating, and sharing information to solve problems (Fetaji, 2021). Thus, it also entailed digital literacy, which included a wide range of skills in using social networking sites and mobile applications, creating electronic content, and understanding cybercrime and safety issues. Developing digital skills is often viewed as encouraging participation in digital life safely and effectively (Fetaji, 2021). The study shows that training on digital skills is, in most cases, significant to help individuals identify the resources that best suit their needs (Titan et al., 2021). The ubiquitous use of digital technologies may lead to changing relations in social life and open new avenues for higher education institutions to ensure the innovation, digitization, exploration, and dissemination of new knowledge in society. This may also provide impetus to sustain innovation and support for scientific talents, research, management skills, and knowledge transfer (Magni et al., 2021; Titan et al., 2021).

Warschauer and Matuchniak (2010) talked about the effects of information technology in real-life, especially when they linked the interactive parts of speech with the archival parts of writing. This made it possible for many people to talk to each other, no matter where they were or what time it was. It also lets people participate in the social editing of texts, helps people learn more, and makes it easier to make and share content in speech and writing. Na and Chia (2008) looked at parents of 0–6-year-old children to see how informal learning from online resources could change their confidence in parenting and understanding of their children’s development. They saw that parents who used online resources could figure out their children’s linguistic and emotional needs and trust their ability to help with the child’s cognitive, behavioral, and emotional development (Na and Chia, 2008). Digital skills may also help families who have children with chronic diseases or neurological or developmental syndromes like autism to find helpful information and solutions on the Internet as a safe outlet to alleviate stress, anxiety, and depression (Douma et al., 2021).

From an economic and environmental point of view, learning through digital skills provides material possibilities. Learning can also be marketed to other countries and cultures that need these educational stimuli to master new learning courses (Alvarado et al., 2021). So, learning based on digital skills, whether for parents or children, saves waste and helps export learning by making it informal and focusing on providing studying as a service outside the boundaries of study in the Kingdom of Saudi Arabia (Muhammad and Khan, 2021).

## Research questions

COVID-19 pandemic has created the largest disruption ever on education systems, affecting nearly 1.6 billion learners in more than 190 countries across the world. Many schools and other learning centers closed that influenced 94 percent of the world’s student population, up to 99 percent in low and lower-middle income countries (Burkadze, 2022).

The emergence of the Corona epidemic in its first, second, and third waves led to the complete or partial closure of schools and educational institutions at all levels. Online teaching and assessment approved during this period spelled the development of learners’ abilities to create digital content and parents’ involvement in teaching their children digital learning skills or identifying new motivational skills that were rarely used during physical classes. Thus, this study aimed to identify how parents dealt with their children during the pandemic and how they used their educational and digital skills to teach and monitor their education at home. The study summarized in the following questions:

1. What were parents’ digital skills used to teach children at home during the spread of the Corona epidemic?
2. What were the differences in digital skills between parents and their employment status, age, and responsibility in teaching children?

## Theoretical framework

### The concept of digital skills

The combination of knowledge, skills, and attitudes is essential for an individual’s self-realization, development, citizenship, social integration, and employment (Jashari et al., 2020). The core digital skills, however, include the critical use of information technology for work, entertainment, and communication (Andriole, 2018). Supported by communication technology, digital skills generally refer to information retrieval, evaluation, storage, production, presentation, exchange, communication, and participation in collaborative networks over the Internet (Law et al., 2018). Digital skills or digital literacy also mean using digital technologies to access, manage, understand, integrate, communicate, evaluate, and create information safely and appropriately for work (Jashari et al., 2020). Digital skills, or twenty-first-century skills, including problem-solving, digital citizenship, cooperation, and communication, are essential to entering the labor market successfully (Van Laar et al., 2017). Digital skills can also be cognitive, which you can get by reflecting on what you know, or practical, which you can get by using methods and getting experience. Practical skills that include autonomy or independence are learned via self-experience and self-directed activities, and participatory skills are learned via collaborative, directed, and team-led learning processes.

Whereas Van Deursen et al. (2014) identifies a series of three general types of skills:

A. Automation skills include the operational manipulation of technology that is used for operating hardware and software and dealing with networks. Van Dijk (2006) recognizes digital skills such as information handling and content creation as the key to fully acquiring new technologies and software for coexistence, entertainment, and learning in a knowledge society; These skills include handling information and content creation (Van Laar et al., 2017).
B. The cognitive structure of information and the ways to make cognitive schemas that can be used to solve problems are examples of structural skills. They are information-functional skills that include the ability to find, process, and evaluate information.
C. Strategic skills mean the ability to use resources to achieve an objective that includes a willingness to proactively search for information to make information-based decisions.

Strategies for lifelong learning need to address the growing need for advanced digital skills in all jobs and for all learners, including those who grew up with technology and older people. This is important because it helps fill in the gaps in teaching and learning in all fields of science and technology. Digital skills start with ICT integration and learning sooner (Van Laar et al., 2017). Using it more critically and creatively mean investing in human capital to benefit from knowledge economics, with an emphasis on privacy, security, and safety levels (Jashari et al., 2020; Van Laar et al., 2017).

### Factors causing the need for DS

Sunita (2020) identified the following challenges to promoting digital skills:

1. Limited internet availability and limited access to devices: Online platforms may be the only way to reach learners during the closure, but teachers and students reported slow internet and connectivity problems interfering with the seamless flow of learning.
2. Poor infrastructure hampered the design development of attractive and appropriate content suitable for the various levels of learning and different ages of the learner via the Internet.
3. Online learning is not about throwing the book at the learner, nor should the scientific material be reformulated, compiled, and adapted to the needs of the educated audience, which ensures that attention and their interests are captured and preserved.

### Types of digital skills

1. Technological skills: The Internet has helped access the knowledge, thaphics, static or animated images, video, and audio to enable parents to follow their children’s learning paths. It also involved parents in personal learning to improve their awareness of their educational responsibilities for their children and follow-up (Na and Chia, 2008). Educational technologies during the pandemic helped compensate for the teacher’s physical absence. They enhanced the learner’s educational experiences, helped verbal and nonverbal communication through video learning and gestures, and obtained supportive feedback, whether with materials and scientific resources that provide opportunities for student participation and motivation. This also made parents more satisfied with the levels of learning of their children (Daugvilaite, 2021). The platforms have also stimulated online music learning that required participation in playing, visual reading and auditory skills to meet training difficulties. It also helped to analyze the auditory skills of melodies and tones in more detail (Pike and Shoemaker, 2013; Rutkowski et al., 2021).
2. Cybersecurity Skills: It is divided into two parts: (a) Personal security skills: When parents have digital questions and dilemmas, they frequently turn to a few sources of support and advice (Livingstone et al., 2018). The adoption of educational technologies supports learning and assessment, providing general satisfaction to parents and guardians and the ability to quickly adapt their children to these educational applications (Ocaña-Fernández et al., 2019). (2) Information security skills: Often because of this stage, some psychological traits are generated in parents, including application anxiety and technophobia, and this may be due to the forced use of technology during the epidemic (Van Dijk, 2006).
3. Critical skills: They help self-criticism and social withdrawal from contexts that cause a crisis in adapting to reality, such as searching for solutions to the child’s troubled behaviors and improving relations between the child’s interactions with parents. Parents were required to monitor their children’s education and behavior, which the teacher would otherwise take care of in regular school (Douma et al., 2021).
4. Virtual environments also help competitive learning by improving the design of learning materials. (Skulmowski and Xu, 2021) These materials were made with learners’ cognitive abilities in mind so that knowledge could be used and remembered for a long time. This skill requires making informed judgments about the quality and accuracy of the information and knowledge to be produced. Information is evaluated for its validity and value and needs theoretical and practical justifications for acceptance or refusal (Van Deursen and Van Dijk, 2014). The evaluation also depends on the complexity of the information and the tasks to be criticized (Chen, 2013).
  Also, we can judge information well if we fully understand what it says or means behind the words. Information with a lower cognitive load and density is relatively easier to evaluate, and ideas unrelated to digital content lead to cognitive load (Kilic, 2014). Interactive digital learning environments are easier on the brain and promote deep learning, motivation, realism, and fluency. The interaction is also more hands-on, which makes it easier to learn concepts. This, in turn, could develop higher thinking skills of students and their parents, such as reasoning, reflective thinking, critical thinking, and creative thinking (Skulmowski and Xu, 2021).
5. Information skills: During the pandemic, the internet has helped parents navigate economic and social problems and inequality in accessing formal learning by creating learning opportunities and facilitating learning experiences as an alternative to tutoring by browsing different educational websites and different online study groups (DiSalvo et al., 2016).
  Parents’ motive for using the Internet is also to search for information to understand health problems in virtual platforms and read suggestions and comments from parents in cases like their children, to improve their knowledge of the disease and quality of life (Camden et al., 2016). Parents also looked for textual content and related images for clarity and tried to make sense of information with real-life and daily applications in the real world, increasing information navigation and knowledge sharing among colleagues and the public (Chen, 2013). Parents may sometimes push to use artificial intelligence (AI) for accurate knowledge (Ocaña-Fernández et al., 2019). AI may also help parents with their children’s learning through more realistic and diverse applications for children that rely on sound and visual effects compatible with the child’s cognitive development (Lutz, 2019; Ocaña-Fernández et al., 2019).
6. Communication skills: It is defined as the ability to encode and decode messages to build meaning and understand and exchange information in all interactive applications (Van Deursen and Van Dijk, 2014). It is divided into two parts: automatic communication skills (visual and auditory), and alternative communication skills (skills like real-life skills) (Chen et al., 2021; Lappan et al., 2020).
7. It refers to the use of aspects of the environment and manipulation of sensory input variables, where learning can reach higher levels than available by navigating spatial contexts to study abstract knowledge, as it provides accurate representations of learning stimuli in three-dimensional electronic environments (Kuhrt et al., 2021; Koehorst et al., 2021). Navigating knowledge is a tool for linking the skills of the twenty-first century, which are technology, information management, communication, collaboration, creativity, critical thinking, problem-solving, and contextual skills for learning, including cognitive flexibility, cultural awareness, moral awareness, self-direction, and lifelong learning (Van Laar et al., 2017). Also, parents’ mastery of digital skills varies according to different social strata (Van Deursen et al., 2014; (Skulmowski and Xu, 2021).

## Method and procedure

### Methodology

To analyze the extent to which parents and guardians mastered digital skills and knowledge development during COVID19, the study used the descriptive-analytical approach.

### Design

The study employed a questionnaire with three parts. The first part is demographic information about respondents, including gender, education level, age, work state, experience, teaching responsibilities, and work dependency on PC. The second one is about technological skills. The third section is about measuring personal security skills. The fourth is about critical skills, and the fifth is measuring hardware locking skills. The following is about information skills, and the seventh is about communication skills. The last two parts are about knowledge navigation and electronic social skills.

### Participants

Two hundred fifty students’ Saudi parents participated in the study. An available sample has been drawn. The questionnaire items were answered through the Google Forms platform. Everyone was free to withdraw whenever they saw that this measure did not fit their preferences. The study classified demographic variables as in Table 1:

**Table 1:** Demographic variables description.

Variables	Levels	Frequencies	Percent
Gender	Fathers	183	73.2%
	Mothers	67	26.8%
Education level	Diploma	41	16.4%
	Bachelors	132	52.8%
	Post-graduate study	3	1.2%
	Master’s degree	39	15.6%
	Ph.D	35	14%
Age level	20–30 years	14	5.6%
	30–40 years	83	33.2%
	40–50 years	114	45.6%
	More than 50 years	39	15.6%
Work state	No job	38	15.2%
	Has a job	212	84.8%
Experience years	Less than 5 years	24	9.6%
	5–10 years	29	11.6%
	10–15 years	49	19.6%
	More than 15 years	148	59.2%
Teaching responsibilities	fully responsible	75	30%
	Partially responsible	73	29.2%
	Share with father and mother	86	34.4%
	You have no direct involvement in teaching	16	6.4%
Work dependency on PC	Yes	203	81.2%
	No	47	18.8%

## Measures

### Digital skills scale

#### The first stage

The scale aimed to verify the degree of mastery of digital skills for parents during COVID-19 and the extent of prior or current knowledge of these skills through practical work skills. Previous studies that dealt with digital culture, virtual skills, and digital skills have been reviewed, such as (Rodríguez-de-Dios et al., 2016; Van Deursen, et al., 2014; Van Laar et al., 2018; Van Deursen and Van Dijk, 2014). The skills scale is included in Supplementary Appendix 1.

#### The second stage

The study used content analysis to look at digital skills in many fields. It also looked at eight theoretical skills. At the same time, from an empirical point of view, it was confirmed that these are the main three-factor model skills: operational skills, cognitive constructivism skills, and instrumental skills. The study used a five-point Likert scale for collecting responses (1 = does not correspond at all, and 5 = corresponds exactly).

#### The third stage

51 items were distributed over the eight theoretical subscales, and exploratory factor analysis (EFA) was accompanied to verify whether the item factor loadings was regular on the nature of the phenomenon from an empirical view.

## Procedures and data analysis

**Fig. 1:**
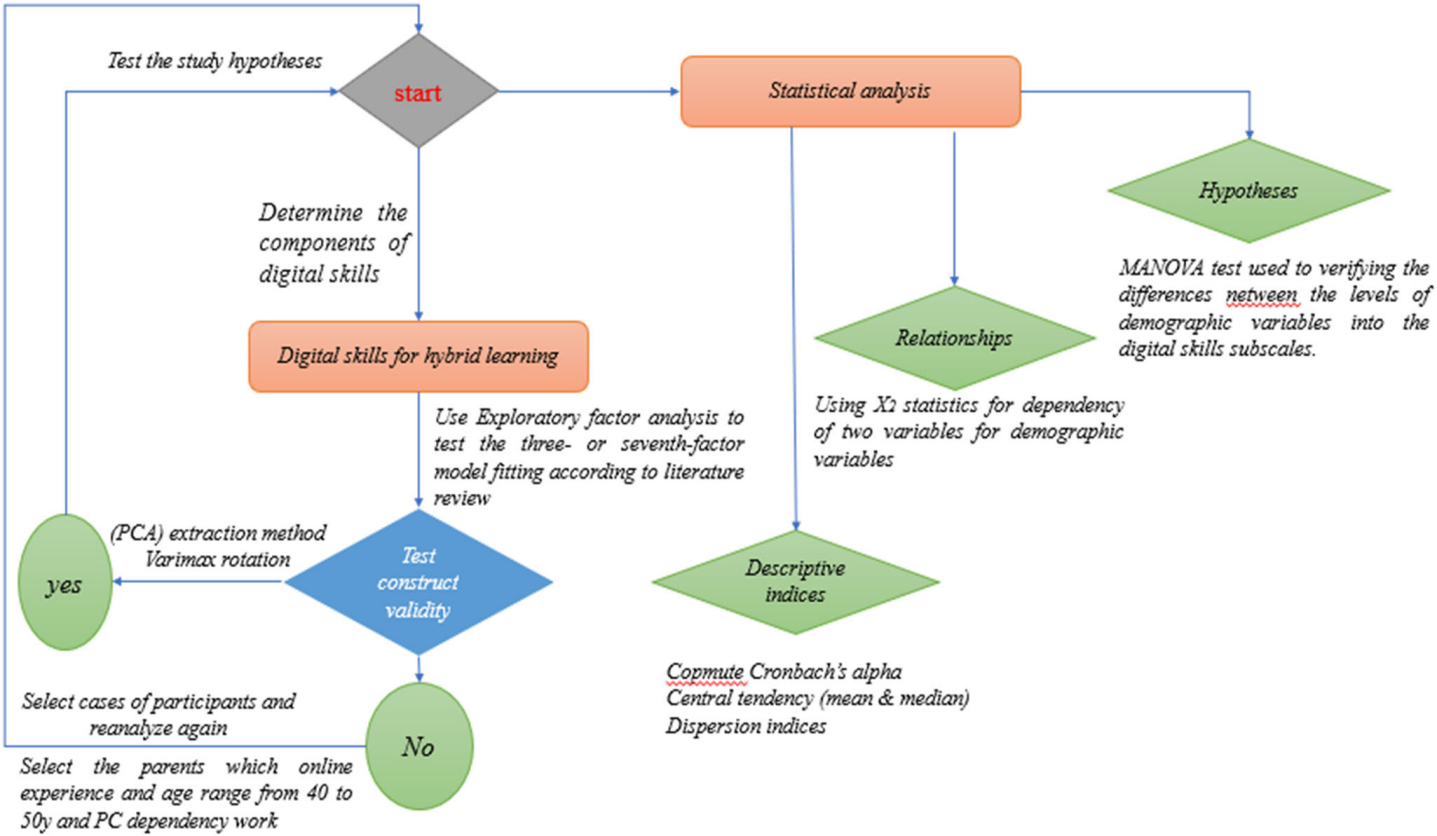
The overall procedures in the study.

Responses on subscales were done by IBM SPSS V.23 software. The cut-score of item factor loadings below 0.50 was rejected. The parsimonious criteria were verified. Parsimonious criteria assumed by saturating each item with only one factor. The principal components analysis (PCA) method was analyzed. The correlation matrix was rotated in the varimax orthogonal rotation. The stability coefficient was performed by Cronbach’s alpha coefficient. The chi-square statistic value of independence of two variables was conducted to examine the associations between demographic variables and the educational responsibility variable of parents towards their children. The MANOVA test was computed to test the differences in digital skills in favor of demographic variables. The following figure shows the overall procedures in the study (Fig. 1).

## Ethical considerations

All procedures performed in the study were following the ethical standards of the institutional research committee of scientific Research Dean of Hail University (IRB Log Number: RG-21-064) and with the 1964 Helsinki declaration and its later amendments.

## Results and discussion

### Content validity

The item analysis was analyzed using EFA, using the PCA method, without determining the number of factors explained which items were organized. In this case, the items can be loaded freely on the dimensions that describe the phenomenon from an empirical view. The Varimax orthogonal rotation was done, given that the resulting factors are independent of each other.

The analysis results reached the KMO criterion value of 0.94, indicating the sample’s suitability and ability to understand what is required to explain the phenomenon under study. The commonality values ranged from 0.4 to 0.75, which means that the items have discrimination between the individual’s performance on the scale.

The results revealed eight factors that could explain the phenomenon before rotation. These factors explained 68.80% of the total variance that explains the phenomenon. The eigenvalues of the analysis were 23.35, 2.86, 2.20, and 1.78. The excess of the underlying eigenvalues approximated about 1.2. The factors’ explained variances were 45.9%, 5.6%, 4.3%, and 2.7%, and the excess of the explained variances neared 2.2%, which is very small in expressing the phenomenon. It was noted that the eigenvalue of the first factor is statistically inflated, which means that this factor is polarizing for the items. In other words, these eight factors are just theoretical factors organized into a more extensive general factor that explains these digital skills. The resulting structure describes the digital skills of the custodian in searching for knowledge, caring for and managing it in the form of texts, and summarizing it to show the knowledge in a coded way.

Statistically expressed, this is what necessitated the use of orthogonal rotation. Since the first factor made the items polarized, the rotation made three factors with eigenvalues of 10.38, 10.19, and 7.85. The total variance explained by the main three-factor model was 55.71%. Statistically, this is logical since digital adequacy does not mean complete mastery of transferring and using knowledge management only, but those digital skills depend on the novelty and strangeness of concepts and the individual’s ability to enrich and apply that knowledge, and the difference in individual differences between individuals in abilities and traits, which critically presents knowledge. The item factor loadings are as in Table 2:

**Table 2:** Item factor loading of digital skills subscale.

Items	Item factor loading		
	Factor (1)	Factor (2)	Factor (3)
Technological skills			
1		0.67	
2		0.59	
3		0.66	
4		0.57	
5	–	–	–
6			.53
7		0.65	
8*		0.54	
9		0.60	
10		0.55	
11		0.66	
Personal security skills			
12		0.51	
13	0.54		
14	–	–	–
15	0.64		
16	0.63		
17	0.65		
Critical skills			
18	0.57		
19	0.61		
20	0.65		
21	–	–	–
22	0.62		
Hardware locking skill			
23	0.68		
24	0.77		
25	0.62		
26	0.81		
27*	0.71		
28*	0.73		
Information skills			
29	–	–	–
30	–	–	–
31		0.56	
32		0.60	
33		0.61	
34*	0.62		
Communication skills			
35			0.68
36			0.63
37*			0.57
38	–	–	–
39*		0.65	
40	–	–	–
41	0.57		
Knowledge navigation			
42	0.53		
43		0.65	
44		0.65	
45		0.59	
46		0.60	
Electronic social skills			
47			0.54
48			0.66
49			0.70
50			0.67
51			0.72
Eigenvalue	10.38	10.19	7.85
Explained variance (%)	20.34%	19.98%	15.39%
Alpha coefficients	0.95	0.95	0.91
Mean			
Std deviation			

(*) means revised items.

The items’ factor loadings for personal, critical, and hardware security skills on a single factor for operational skills are shown in the table above. This factor says that operational skills describe the security of information, data, and devices, as well as the security of an individual.

Confirmatory factor analysis used to test the EFA model of digital skills. The results as the followings (Table 3):

**Table 3:** Digital skills model goodness of fit.

Index	RSMEA	GFI	NNFI	PNFI	SRMR	*X*^2^
Value	0.11	0.92	0.95	0.90	0.069	5718.4*

*Means the significance of x^2^ (df) as a bad index for the digital skills model. The x^2^ (df) index is sensitive to the normality of data and sample size.

The results of the confirmatory factor analysis resulted in good fitting indicators, except for the chi-square and the RMSEA indices, which went out of range, and this is due to the violation of the multivariate normality indicated by the LISREL program in the statistical analysis. The item factor loadings as Table 4:

**Table 4:** Item factor loading of digital skills subscale.

Items	Factor loadings	Std error	*t*-value
CCS1	0.63	0.043	14.73
CCS2	0.61	0.043	14.15
CCS3	0.70	0.042	16.75
CCS4	0.61	0.043	14.14
OPS6	0.64	0.043	14.95
CCS7	0.75	0.040	18.50
CCS8	0.71	0.041	17.18
CCS9	0.77	0.040	19.11
CCS10	0.71	0.041	17.30
CCS11	0.74	0.041	18.06
CCS12	0.64	0.043	14.97
ES13	0.69	0.042	16.45
ES15	0.64	0.042	15.18
OPS16	0.75	0.040	18.40
OPS17	0.73	0.041	18.04
OPS18	0.74	0.041	18.17
OPS19	0.70	0.041	16.82
ES20	0.73	0.041	17.75
ES22	0.77	0.040	19.30
ES23	0.68	0.042	16.35
ES24	0.78	0.040	19.64
ES25	0.71	0.041	17.18
ES26	0.80	0.039	20.43
ES27	0.83	0.039	21.51
ES28	0.81	0.039	20.68
CCS31	0.56	0.044	12.73
CCS32	0.79	0.039	20.18
CCS33	0.76	0.040	18.90
ES34	0.82	0.039	21.06
OPS35	0.84	0.039	21.89
OPS36	0.82	0.039	20.86
OPS37	0.54	0.045	12.14
CCS39	0.79	0.039	20.01
ES41	0.81	0.039	20.72
ES42	0.77	0.040	19.14
CCS43	0.83	0.038	21.61
CCS44	0.75	0.040	18.67
CCS45	0.70	0.040	16.83
CCS46	0.79	0.039	20.03
OPS47	0.82	0.039	20.98
OPS48	0.77	0.040	19.14
OPS49	0.80	0.040	20.12
OPS50	0.60	0.044	13.62
OPS51	0.73	0.041	17.74

*OPS* operational skills, *ES* elementary skills, CCS cognitive constructivism skills.

All vocabulary saturations were statistically significant on all dimensions. The saturations on the scale ranged from 0.56 to 0.84, which are medium-to-high saturations, which indicate the reliability of the scale and its relevance to the nature of the sample. The items’ factor loadings for personal, critical, and hardware security skills on a single factor for operational skills are shown in the table above. This factor says that functional skills describe the security of information, data, and devices, as well as the security of an individual.

The reliability coefficient was performed by Cronbach’s alpha and equaled 0.954, and the items’ reliability coefficients ranged from 0.950 to 0.954.

It is clear from the results that technological skills, informational skills, and knowledge navigation skills can be called cognitive constructivism skills. Although technical skills depend on programs and applications, they are concerned with knowledge, whether searching for it or treating it. Cronbach’s alpha had a reliability coefficient of 0.949, and the other reliability coefficients were between 0.945 and 0.948 .

The social and technological skills and the first three items of the communication skills are saturated in one dimension that can be named after the automated skills. The first item concerns the automated technological aspect in interactions or the production or output of texts. It was pointed out that the communication skills dimension is unstable and that its items are split into two dimensions, which is different from the automated skills dimension. Cronbach’s alpha showed that the reliability coefficient was 0.911, and the reliability coefficients ranged from 0.893 to 0.911.

### Descriptive statistics for the study

The descriptive statistics indices were estimated for the digital skills subscale such as mean, median, standard deviation, variance, and skewness, and the results were as in the Table 5:

**Table 5:** The descriptive indices for digital skills subscale.

	Elementary skills	Cognitive constructivism skills	Operational skills
Mean	66.89	36.98	65.04
Median	67	38	64
Std deviation	14.02	6.37	14.21
Variance	196.64	40.52	201.83
Skewness	–0.23	–0.36	–0.19

**Fig. 2:**
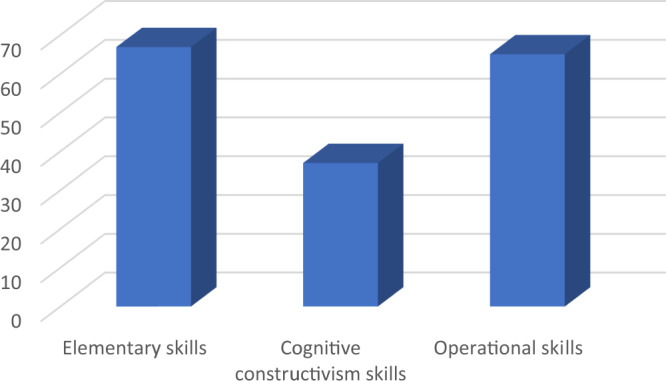
The average value of digital skills subscales.

By using frequencies, the results showed that there were no outliers in the data. The score variance was higher than the mean index, which means there was a massive variance in individuals’ skills. It was noted from the rates of torsion that the individuals’ degrees follow the average distribution. The Fig. 2. Showed the digital skills subscales mean:

Shapiro- Wilk test performed to test the normality of the dependent variables. The elementary skills were non- normal data (statistic = 0.971, *P* = 0.000). the Cognitive constructivism skills has no normal data (statistic = 0.976, *P* = 0.000). Operational skills were no normality (statistics = 0.927, *P* = 0.000).

### The relationship between children’s teaching responsibility and experience

Chi-square test statistic for the two variables’ independence was performed to examine the association between the adoption of teaching responsibility for children in hybrid learning, depending on the years of experience of the parents. The results revealed statistical significance (*X*^2^ = 26.16, df = 9; *P* = 0.002). The teaching responsibility of teaching children during the COVID19 relied on experience with digital skills.

### The relationship between the teaching responsibility of the children and the dependence of the parents’ job on use of the computer

The chi-square results were not statistically significant (*X*^2^ = 2.53, df = 3; *P* = 0.470). It means that the participation of parents in the educational responsibility of teaching their children in the conditions of the epidemic does not depend on the parent’s reliance on the use of the computer, and therefore this means coexisting and adapting to the conditions of the epidemic. It means that the experience received by the learner occurred during the teacher’s guidance. The learner receives his grades continuously after being subjected to a typical assessment process, and therefore there is no effect on the nature of the parents’ profession being linked to computer skills. Support may be received through the internet for children with special needs in the form of instructions or assignments performed by parents.

### The relationship between the educational responsibility of the children and the educational level of the parents

The chi-square test showed that there were statistically significant (*X*^2^ = 26, df = 12; *P* = 0.011). This means that the parental teaching responsibility during a pandemic is associated with the parent’s educational level. In the sense that the digital skills mastered through education may help the child to advance in the learning process, as it acquires higher-order thinking skills by training his parents.

### The relationship between the educational responsibility of the children and the age of the parents

The results of the chi-square statistic between the adoption of the teaching responsibility of the children and the chronological age of the parents were not statistically significant (*X*^2^ = 10.33, df = 9; *P* = 0.325). The result implications that the participation between parents’ responsibility in the teaching during the epidemic is not dependent on the parent’s age.

### Differences in digital instrumental skills are due to the parent’s gender, employment state, age, and responsibility for teaching children

The study used the multiple analysis of variance (MANOVA) test to determine the differences in the instrumental skills of parents during the ongoing Corona pandemic. The results revealed the following in Table 6:

**Table 6:** Differences in digital instrumental skills according to demographic variables.

Source		Sum of squares	df	Mean square	*F*	Sig.
intercept	Hypothesis	31745.553	1	31745.553	94.889	0.000
	Error	3961.928	11.842	334.554^a^		
status	Hypothesis	7.410	1	7.410	0.050	0.823
	Error	34195.848	231	148.034^b^		
education level	Hypothesis	967.068	4	241.767	1.633	0.167
	Error	34195.848	231	148.034^b^		
age level	Hypothesis	1816.478	3	605.493	4.090	0.007
	Error	34195.848	231	148.034^b^		
job status	Hypothesis	323.993	1	323.993	2.189	0.140
	Error	34195.848	231	148.034^b^		
experience	Hypothesis	1168.682	3	389.561	2.632	0.051
	Error	34195.848	231	148.034^b^		
kind_of_job	Hypothesis	924.456	2	462.228	3.122	0.046
	Error	34195.848	231	148.034^b^		
instructional responsibilities	Hypothesis	4102.509	3	1367.503	9.238	0.000
	Error	34195.848	231	148.034^b^		
depend_on_PC	Hypothesis	1293.756	1	1293.756	8.740	0.003
	Error	34195.848	231	148.034^b^		

^a^The error refers to the variance that occurred by variance between groups.

^b^The error refers to the variance that occurred by variance within groups.

The results concluded that there were no differences between parents in digital instrumental skills due to the ongoing Corona pandemic, and the mastery of these skills was not affected by the parents’ academic levels. These skills upgraded from a minor elementary skill to educational adequacy. In addition, age affected this skill, and this can be explained in two ways:

1. Age is an influential index in mastering skills due to the hybrid learning conditions during pandemic situations. Or, through the individual’s pursuit of non-formal education to achieve educational competencies for continuous learning.
2. Younger parents are more proficient in these skills, as their combination of social media and other software is behind this proficiency in the use of technology.

The study considers the second proposition to be logical. The value of the non-indicative effect of the number of work experience years justifies this. Furthermore, mastering technology and machine skills is not a requirement imposed on the elderly based on years of work experience. The nature of the work (governmental, private) requires training in these skills. Then, the confidential work imposes a set of competitive advantages between institutions. Therefore, the individual constantly attempts to achieve better levels of proficiency, which may have happened because of the dependency on computers.

### The differences in cognitive constructivism skills are due to the parent’s gender, employment status, age, and teaching responsibilities in teaching

The study depended on MANOVA to compute the differences in the cognitive constructivism skills of parents during the ongoing Corona pandemic. The results identified in Table 7:

**Table 7:** Differences in cognitive constructivism skills according to demographic variables.

Source		Sum of squares	df	Mean square	*F*	Sig.
Intercept	Hypothesis	30426.815	1	30426.815	77.782	0.000
	Error	3021.866	7.725	391.180^a^		
status	Hypothesis	475.017	1	475.017	3.342	0.069
	Error	32834.827	231	142.142^b^		
education level	Hypothesis	4829.726	4	1207.432	8.495	0.000
	Error	32834.827	231	142.142^b^		
age_level	Hypothesis	1476.885	3	492.295	3.463	0.017
	Error	32834.827	231	142.142^b^		
job status	Hypothesis	447.493	1	447.493	3.148	0.077
	Error	32834.827	231	142.142^b^		
experience	Hypothesis	515.097	3	171.699	1.208	0.308
	Error	32834.827	231	142.142^b^		
kind_of_job	Hypothesis	173.948	2	86.974	0.612	0.543
	Error	32834.827	231	142.142^b^		
instructional responsibilities	Hypothesis	1638.624	3	546.208	3.843	0.010
	Error	32834.827	231	142.142^b^		
depend_on_PC	Hypothesis	1997.764	1	1997.764	14.055	0.000
	Error	32834.827	231	142.142^b^		

^a^The error refers to the variance that occurred by variance between groups.

^b^The error refers to the variance that occurred by variance within groups.

The results agreed on the significance of each of the effects of cognitive constructivism skills depending on the age, education levels, the educational responsibility of the parent towards their children, and the computer skills-based work. This is maintained by several reasons, including:

1. The individual’s ability to formulate knowledge in models, figures, schemas which facilitates children skipping the stage of education during the ongoing epidemic.
2. Parents’ dependence on alternative digital sources in searching for information, formulating knowledge, managing it, and criticizing. It may have helped the learner to achieve a specific educational goal.
3. The learner can reach the cognitive level in a more flexible manner, which helps him achieve learning goals.
4. Parents’ ability, throughout life, is to know their children’s knowledge requirement, transfers to the learner the skills of the codified search for rich sources of knowledge.
5. The ability to navigate knowledge because of mastering computer skills and, of course, because of practicing different technologies and software, made it an automated process in the search for knowledge.

### Differences in operational skills are due to the parent’s gender, employment status, age, and responsibility for teaching children

MANOVA results tested the differences in the operational skills of parents during the ongoing Corona pandemic. The results reached as in Table 8:

**Table 8:** Differences in operational skills according to demographic variables.

Source		Sum of squares	df	Mean square	*F*	Sig.
Intercept	Hypothesis	10974.469	1	10974.469	182.453	0.000
	Error	563.970	9.376	60.150^a^		
status	Hypothesis	46.557	1	46.557	1.381	0.241
	Error	7789.755	231	33.722^b^		
education level	Hypothesis	614.295	4	153.574	4.554	0.001
	Error	7789.755	231	33.722^b^		
age level	Hypothesis	226.674	3	75.558	2.241	0.084
	Error	7789.755	231	33.722^b^		
job status	Hypothesis	161.406	1	161.406	4.786	0.030
	Error	7789.755	231	33.722^b^		
experience	Hypothesis	109.897	3	36.632	1.086	0.356
	Error	7789.755	231	33.722^b^		
kind_of_job	Hypothesis	66.117	2	33.059	0.980	0.377
	Error	7789.755	231	33.722^b^		
instructional_responsibilities	Hypothesis	468.273	3	156.091	4.629	0.004
	Error	7789.755	231	33.722^b^		
depend_on_PC	Hypothesis	19.867	1	19.867	0.589	0.444
	Error	7789.755	231	33.722^b^		

^a^The error refers to the variance that occurred by variance between groups.

^b^The error refers to the variance that occurred by variance within groups.

According to the previous table, the parents’ educational level, work in a specific job, and teaching responsibility to the children during the pandemic created an incentive to work with them in a way that enabled them to achieve their learning goals. Perhaps the performance goals and the mastery goals improved among the children because of the parents’ efforts to improve the assignments required of the children. The presentation, interrelationship, management, and summarization of the learning contexts may have contributed to achieving children’s learning goals, and the operational skills of the parents may also have created perceived pleasure in learning for the children.

## Discussion and delimitations

The study aimed to verify the digital skills of parents of students in Saudi society, considering the Corona pandemic. The study made a scale with eight theoretical dimensions that were based on the results of other studies. Moreover, it was verified empirically and reached three dimensions approved by some previous studies (Van Deursen et al., 2014).

The results concluded that there was a discrepancy in the performance of the sample, which was very high in the operational skills dimension, instrumental skills, and finally cognitive constructivism skills. The reason for the low variance among participants can be explained that parents had benefited from the same amount of knowledge in solving problems. Either information processing processes may have reduced the level of the learner’s cognitive load in acquiring knowledge, which causes high mastery or achievement performance and permits the performance, and this is logical with the study (Kilic, 2014).

The results also seem logical regarding the existence of digital skills differences due to the teaching responsibility of parents, and this may be due to the main goal, whether for the learner or parents, is to solve educational problems or to provide the appropriate support when it comes to health or education with the ease that individuals proceed to pursue informal learning (Camden et al., 2016; DiSalvo et al., 2016).

There are also differences between the parents’ dependence on the computer in affecting instrumental skills and cognitive constructivism skills. It means that the parents’ familiarity with some digital skills helps them gain experience and find new ways to solve problems more realistically and wisely. The study identified that, where possible, parents used digital platforms with an interest in solving educational problems or to obtain stimuli that act as educational scaffolding that expand the reflective thinking of children as observed elsewhere (Chen, 2013; Ocaña-Fernández et al., 2019; Skulmowski and Xu, 2021).

The results of the study can be applied through online courses to educate parents, especially mothers. Digital skills are cultural learning frameworks that are compatible with traditional learning. Digital skills facilitate the learner to generate content that may work as a transition between knowledge, increased knowledge structure, and deeper knowledge representations. The study also identified some limitations. The participants in the study sample consisted of a higher number of fathers than mothers, which makes for a type I error in the cross-validation of the study results. The study also included 65% of participants over 40 years old in terms of experience in raising children may be behind the 55% explained variance of the three factors of the digital skills scale. The unexplained variance may be because of the sample identified by its wisdom in the creasing age. The participants’ responses suffered from social approval because of the large sample size or the participants’ feeling of low social self if they responded logically on the scale, which justifies the highest inequalities in instrumental skills and operational skills.

### Limitations

The study results can be generalized to parents, especially those aged 30 to 50 years, and families with students at the university, secondary, and preparatory stages, as the learner can use his parents as mediators in directing him to the stimuli of learning. Parents’ digital skills also provide avenues for learning outside of the classroom. It also lets the learner take courses through MOOCs to get deeper knowledge in different areas of education, especially at the post-graduate level. It is possible to have parents with low educational levels in digital skills search for and navigate knowledge methods to improve the standards of the cognitive structure associated with digital skills. Also, when parents are involved in their children’s education plans, it makes learning more satisfying for both the parents and the children. When parents keep an eye on their children, the children become more responsible.

### Educational applications

The school establish WhatsApp groups for parents meeting to communicate, change with them points of view concerned to students’ performance, their adaptation of rules, provide safe environment for their children. These meeting can be solved problem to help them to overcome the problem or difficult they face while dealing with digital learning.

## Supplementary information

digital skills scale

## Data Availability

Data are available in Figshare: 10.6084/m9.figshare.21836058.v1.
