# Supplementary material for: Parents’ digital skills and their development in the context of the Corona pandemic
Source: Humanit Soc Sci Commun. 2023 Mar 11;10(1):97. doi: 10.1057/s41599-023-01556-7 (PMC10005916; doi:10.1057/s41599-023-01556-7)
Supplement: Supplementary file 1 — digital skills scale [file 41599_2023_1556_MOESM1_ESM.docx]

**Appendix: Digital skills scale:**

1. Your responsibility to teach children during the Corona pandemic
2. Your job depends on your computer skills
3. I can save sites I like so I can watch them later (using a bookmark....).
4. I can save the image that I find on the Internet in the appropriate extension Jpg, PDF, PNG, TIFF and gif
5. I can download information/files that I find suitable from the Internet for my interests.
6. I use keyboard shortcuts.
7. I find it difficult to deal with smartphone applications related to information management, preservation and representation.
8. I can install or remove programs and applications according to my needs.
9. I can process images by specialized software.
10. I have the ability to use specialized websites for self-learning.
11. I can create spreadsheets using spreadsheet programs.
12. I deal with e-learning platforms (My Platform - Coursera - Model - Blackboard.) to easily manage the various learning processes.
13. I have the ability to record and process digital audio using an audio application.
14. I can control the opening or closing of geolocation in social media
15. I can report abuse on social media if someone uses my information without my permission.
16. I can change my social media sharing settings to choose who can see my posts
17. I know the consequences of illegal downloading music and video content from the Internet
18. I compare different apps to choose the most reliable and not the one. (I download the most reliable apps)
19. Choose a strong password (difficult to know) to save important files
20. I compare different sources to see if the information about me is correct.
21. I determine if information I find online is reliable.
22. If I tell someone online I know how to check if their profile is real
23. I find it difficult to determine how useful the information is for my purposes
24. I have confidence in my assessment of whether a website can be trusted
25. Use antivirus software to detect and remove viruses
26. I can identify malicious files.
27. I block unwanted spam.
28. I have the ability to know any malfunctions that may occur when I use my personal device or smartphone.
29. I can manage passwords on websites and applications in a secure way.
30. I can synchronize my smart devices with simultaneous mapping of all the information
31. I'm having a hard time deciding the best keywords to search online.
32. It's hard for me to figure out the programming language in which many websites are designed.
33. I can use document preservation programs for books or scientific references such as Mendeley; Endnote, …..
34. I can identify the type of electronic files by knowing the file extension (doc - exe - ppt - mp3…).
35. I can visualize information using presentation software or applications.
36. I have the ability to make backups of important files to a specialized drive or place.
37. I can make a call or call online (through social media programs) to whomever I want.
38. I have the ability to send e-mail with its various attachments (images - video - texts - audio - ...).
39. Use social networking programs (WhatsApp - Messenger - ...) to send important messages.
40. I connect all the social media applications and websites that I subscribe to with my smartphone to access the different contacts.
41. I can share large files on specialized sites.
42. I can manage my profile on the electronic educational institution’s platforms.
43. I can work remotely on shared files (such as shared and uploaded files to Google Drive).
44. I know how to use a wide variety of strategies when searching for information.
45. I compare different websites to determine the quality of services each website provides.
46. I know how to open a website address directly without using a search engine like Google.
47. I have no problems finding information whatsoever in the website.
48. I can choose and use smart search engines that are appropriate for the subject area I am searching for.
49. I strictly limit the information that can and cannot be shared online.
50. I am careful to make my comments and behavior appropriate to the situation I find myself in online.
51. I have the ability to select the people I share content with (eg friends, friends of friends, family...).
